# Ethnobotanical investigation on medicinal plants in Algoz area (South Kordofan), Sudan

**DOI:** 10.1186/s13002-018-0230-y

**Published:** 2018-04-27

**Authors:** Tahani Osman Issa, Yahya Sulieman Mohamed, Sakina Yagi, Reem Hassan Ahmed, Telal Mohammed Najeeb, Abdelrafie Mohamed Makhawi, Tarig Osman Khider

**Affiliations:** 1 College of Applied and Industrial Sciences, University of Bahri, P.O. Box 1606, Khartoum, Sudan; 2 Institute of Medicinal and Aromatic Plant National Centre for Research, Khartoum, Sudan; 3 Department of Botany, Faculty of Science, University of Khartoum, P.O. Box 11115, Khartoum, Sudan

**Keywords:** Medicinal plants, Healers, Algoz area, Sudan

## Abstract

**Background:**

The inhabitants of western Sudan use traditional medicine for the treatment of various ailments due to lack of medical doctors and unaffordable prices of pharmaceutical products. The present study is the first documentation of the traditional plant knowledge on medicinal uses of plants by healers in Algoz (South Kordofan), Sudan.

**Method:**

Ethnobotanical data were collected over a period from March to November 2015 using semi-structured interviews with 30 healers (24 male and 6 female) living in the investigated area. Quantitative indices such as use categories, use value (UV) and informant consensus factor (ICF) were intended to evaluate the importance of medicinal plant species.

**Results:**

A total of 94 medicinal plants, which belong to 45 families and 81 genera, were recorded in the study area. The most represented families are Leguminosae with 20 species followed by Combretaceae (6 species), Rubiaceae (5 species) and Asteraceae (4 species). The reported species were belonging to herbs (43%), trees (28%), shrubs (22%), climbers (4%) and parasites (3%). Root and stem (21% each) were the most plant parts used. A majority of remedies are administered orally (67%) where infusion (36%) and maceration (32%) are the most used methods. The highest ICF (0.87) was reported for poisonous animal bites followed by urinary system diseases (0.89), blood system disorders (0.88) and gynaecological diseases (0.87). *Anastatica hierochuntica*, *Ctenolepis cerasiformis*, *Echinops longifolius*, *Cleome gynandra*, *Maerua pseudopetalosa*, *Martynia annua*, *Oldenlandia uniflora*, *Opuntia ficus-indica*, *Solanum dubium*, *Sonchus cornutus*, *Tribulus terrestris* and *Drimia maritima* were reported for the first time in this study.

**Conclusion:**

The number of medicinal plants reported in this paper reflects evidence that Algoz area had a high diversity of medicinal plants which will continue to play an important role in the healthcare system in the study area.

## Background

In 2011, Sudan split into two countries with one third of the country being proclaimed a new state named “Republic of South Sudan” leaving behind the remaining area retaining the older name “the Republic of Sudan” [1]. In its former integral state, Sudan was the largest country in Africa and the tenth in the world, boasting an area of 2.5 million square kilometers which spanned diverse terrains and climatic zones [1]. This did bear directly on the wide diversity of vegetation, from those in the desert and semi-desert in the north through the equatorial in the central part to the extreme of the humid equatorial in the south. Such prevailing conditions favoured diverse vegetation consisting of 3137 documented species of flowering plants belonging to 170 families and 1280 genera, 15% of which are endemic [2]. A large number of these plants have a vital contribution to human health care needs throughout the country. Medicinal and aromatic plants and their derivatives represent an integral part of life in Sudan. Communities in different regions of Sudan use traditional medicine for the treatment of various ailments due to lack of medical doctors and unaffordable prices of pharmaceutical products beside their faith on the medicinal values of traditional medicine [3]. It has been estimated that only 11% of the population has access to formal health care [1].

The geographical position of Sudan represents a multicultural melting pot of diverse traditional knowledge over large distances and facilitated the exchange of knowledge about medicinal plants with other countries from Africa to Middle East and Asia [4].

Despite the varied flora and socio-cultural diversity in Sudan, there is a far-reaching lack of written information on the traditional use of medicinal plants [4]. So, documentation of plants used as traditional medicines in Sudan is warranted. The aim of this study was to investigate the traditional plant knowledge on medicinal uses of plants by local healers in Algoz area (South Kordofan), Sudan.

## Methods

### Study area

**Fig. 1:**
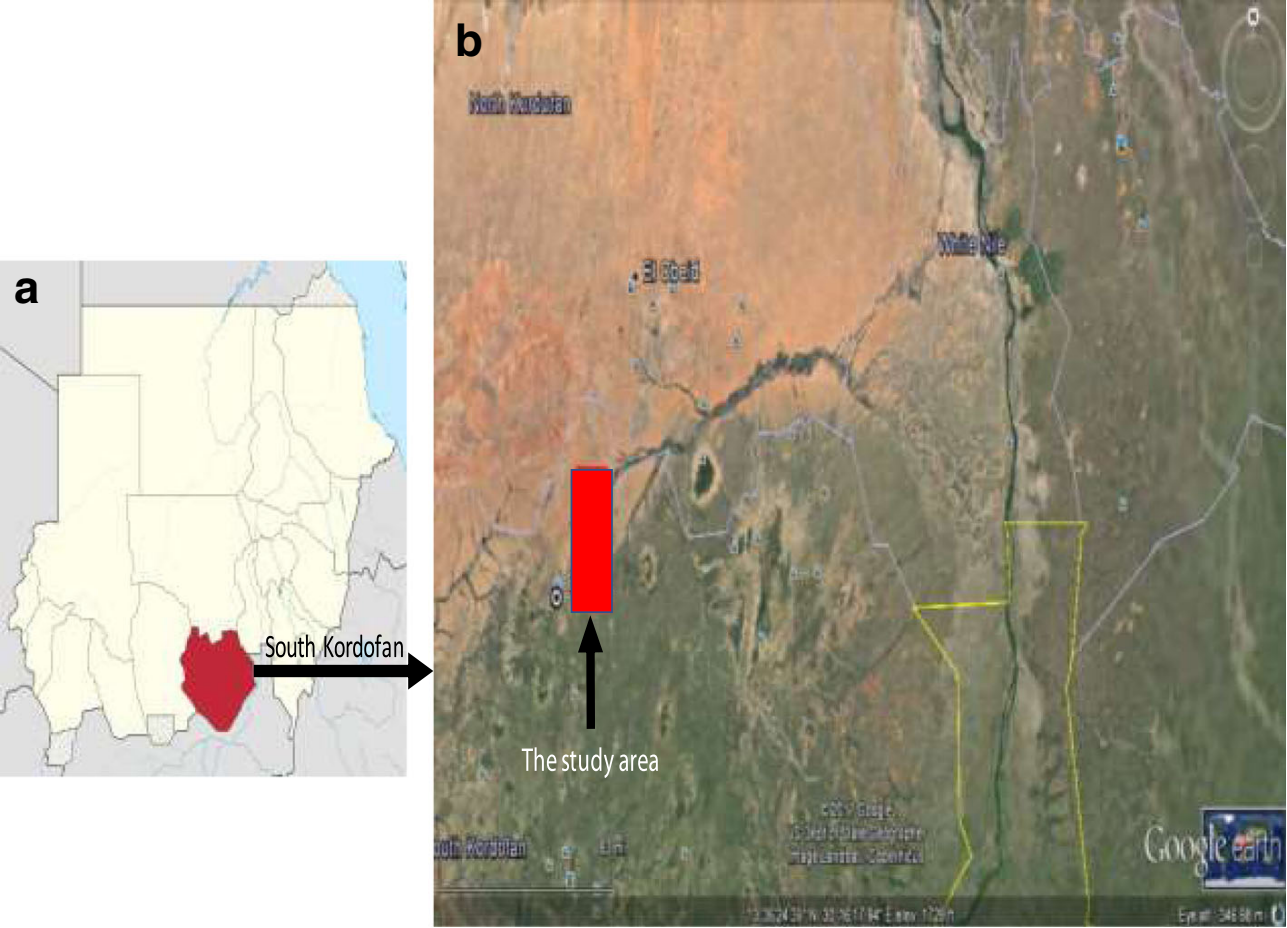
**a** Sudan map showing the South Kordofan State (red) and **b** Algoz locality (red)

Algoz area is situated in the northern part of South Kordofan state, and its borders are Northern Kordofan state from the north and northeast, West Kordofan state from the northwest, Dellang locality from the south and Habella locality from the southeast direction (Fig. 1). It is located between latitudes 12°–12° 30 N and longitudes 29° 48–300 E and 622 m above sea level, with a total area of 35,000 km^2^. Short grass and short scattered trees prevail. The area is associated with exposed rocks crossing the central Sudan forming a surface water divide. The White Nile which is the main tributary of the River Nile bounds the hydrologic system to the east, while the highlands of Kordofan Plateau and the Nuba Mountains bound it to the west and the south respectively. Khor Abu Habil is a major seasonal wadi that crosses the study area and flows from the west to the east. The wadi disappears into the sand dunes a few kilometers before reaching the White Nile. The climate in the area is semi-arid with long hot summers (March–September) and short mild winters (December–February). Seasonal rainfall occurs only during summer (June–September) and varies between 200 mm/year in the north and 450 mm/year in the south [5].

Algoz area has a multi-population with tribes as Dar Shungool, Gaboosh, Dar Bati, Albargo, Albarno, Flata and some Arabic nomads. They are working mainly in agriculture, animal grazing and trade [6].

### Data collection and plant identification

Ethnobotanical data were collected from March to November 2015. Information about the medicinal use of plants was collected by carrying out semi-structured interviews with 30 healers (24 male and 6 female) living in the investigated area. The questionnaire was designed to collect data on (i) local names of the plants, (ii) ailments treated by the plant, (iii) plant parts used, (iv) condition of the plant material (dried or fresh) and (v) modes of preparation and administration. Some social factors like the name, age, occupation and education level of the interviewed person were also recorded. Also, the geographic locality and date of the interview were recorded. Plant specimens were collected for taxonomic identification using keys of written floras such as Broun and Massey [7], Andrews [8–11], Ross [12], Hutchinson and Dalziel [13], Maydell [14] and Elamin [15]. Voucher specimens were deposited at the Herbarium of Institute of Medicinal and Aromatic Plants, National Centre for Research, Sudan (MAPTMR-H). The botanical names and plant families are given according to the standards of the plant list (www.ipni.org/).

### Ethnobotanical data analysis

Data analysis was carried out by using both the classical ethnobotanical systematic investigation and a numerical quantitative approach in order to evaluate the importance of the mentioned plant species in the investigated area. The quantitative study was carried out by calculating the following ethnobotanical indices:

#### Use categories

The medicinal plant uses were classified into categories following the standard developed by Cook [16]. Each time a plant was mentioned as “used” was considered as one “use report”. If one informant used a plant to treat more than one disease in the same category, it was considered as a single use report [17].

#### Use value (UV)

The relative importance was calculated employing the use value [18], a quantitative measure for the relative importance of species known locally:

$$ \mathrm{UV}=\frac{\sum U}{n} $$

where *U*_i_ is the number of use reports cited by each informant for a given species and *n* refers to the total number of informants.

Use values are high when there are many use reports for a plant, implying that the plant is important, and approach zero (0) when there are few reports related to its use. The use value, however, does not distinguish whether a plant is used for single or multiple purposes.

#### Informant consensus factor

To test homogeneity of knowledge, the informant consensus factor was used [19]:

$$ \mathrm{ICF}=\frac{N_{\mathrm{ur}}-{N}_{\mathrm{t}}}{\left({N}_{\mathrm{ur}}-1\right)} $$

where *N*_ur_ refers to the number of use reports for a particular use category and *N*_t_ refers to the number of taxa used for a particular use category by all informants. Informant consensus factor (ICF) values are low (near 0) if plants are chosen randomly or if there is no exchange of information about their use among informants and approach one (1) when there is a well-defined selection criterion in the community and/or if information is exchanged between informants [20].

## Results

### Medicinal plant diversity

A total of 94 medicinal plants, which belong to 45 families and 81 genera, were recorded in the study area. Results provide the following information for each species: scientific name, botanical family, local common name, plant habitat, plant part used, disease treated, route of administration and use value (Table 1). The most represented families are Leguminosae with 20 species followed by Combretaceae (6 species), Rubiaceae (5 species), Asteraceae (4 species), Lamiaceae, Poaceae, Tiliaceae and Zygophyllaceae (3 species each), Apocynaceae, Asclepiadaceae, Brassicaceae, Burseraceae, Cleomaceae, Capparaceae, Malvaceae and Meliaceae (2 species each), and other families were represented with one species each. This dominance of Leguminosae plants is a characteristic of the Sudan flora. The most commonly used species is *Sarcocephalus latifolius* with a UV of 2.07 followed by *Guiera senegalensis* with a UV of 1.87, *Hydnora abyssinica* with a UV of 1.83 and *Geigeria alata* with a UV of 1.67 respectively. Plants that treat three ailments and more (86%) represent the majority, followed by plants that treat single ailments (8%) and those that treat two ailments (6%) respectively.

**Table 1:** Ethnomedicinal plants used in the Algoz region (South Kordofan)/western Sudan

Plant name/family/voucher no.	Local name	Growth habit	Part used	Ailment treated	Mode of administration	UV
*Abrus precatorius* L., Leguminosae, G/106/83	Habat alaroose	Climber	Seed	Spleen problems	Infusion	0.06
*Acacia nilotica subsp. adstringens* (Schum. & Thonn.) Roberty, Leguminosae, G/56/83	Garad	Tree	Fruit	Stomachache	Powder mixed with ajeen and drunk	0.93
				Cold and flu	Smoke fumigant	
				Wounds	Powder mixed with bee wax	
*Acacia oerfota* (Forssk.) Schweinf., Leguminosae, O/49/78	Laoat	Shrub	Stem	Back pain	Roasted twigs put on the back	1.00
			Leaf	Swellings	Poultice applied to swellings	
			Root	Snake bite	Fresh crushed roots rubbed on the place of bite	
				Toothache	Paste with atroon	
*Acacia senegal* (L.) Willd., Leguminosae, G/110/83	Kitir abied	Tree	Gum	Haematuria	Infusion	0.60
				Toothache	Filling tooth cavity with gum powder	
*Acacia seyal* Delile, Leguminosae, M/21/76	Talih	Tree	Heart wood	Rheumatic pain	Smoke fumigant	0.53
*Acanthorrhinum ramosissimum* (Coss. & Durieu) Rothm., Plantaginaceae, W/14/95	Shagart almassas	Herb	Aerial part	Evil eye	Smoke fumigant	0.40
*Adansonia digitata* L., Malvaceae, W/20/95	Tabaldi/fruit gongolaise	Tree	Fruit	Giardiasis	Decoction of the mesocarp	0.70
				Stomachache	Decoction of the mesocarp	
*Albizia anthelmintica* Brongn., Leguminosae, K/11/96	Um takarny/gerfadud	Tree	Bark	Worm expulsion	Infusion	0.33
			Leaf	Wounds	Powder sprinkled in wound	
				Stomachache	Infusion	
				Jaundice	Infusion	
*Allium sativum* L, Amaryllidaceae, Cultivated	Toom	Herb	Bulb	Haemorrhoids	Mixed with *Zingiber officinale* rhizome and applied to the anus	0.07
*Anastatica hierochuntica* L., Brassicaceae, Purchsed	Kaf maryam	Herb	Aerial part	Postpartum	Maceration or infusion	0.37
*Anogeissus leiocarpus* (DC.) Guill. & Perr., Combretaceae, W/1/97	Sahab	Tree	Bark	Toothache	Filling tooth cavity with powder	0.37
				Jaundice	Maceration or infusion	
				Malaria	Maceration or infusion	
*Anticharis senegalensis* (Walp.) Bhandari, Scrophulariaceae, W/15/94	Shagarat alwaram	Herb	Aerial part	Swellings	Poultice applied to swellings	0.03
*Arachis hypogaea* L., Leguminosae, Cultivated	Foul sodany	Herb	Seed	Bilharzia	Maceration	0.033
*Aristolochia bracteolata* Lam., Aristolochiaceae, G/7/84	Um galagil	Herb	Aerial part	Malaria	Infusion	0.47
				Ear infection	Smoke fumigant	
				Headache	Infusion	
*Azadirachta indica* A. Juss., Meliaceae, W/95/94	Neem	Tree	Leaf and wood	Rheumatic pain	Maceration and taken as bath	0.43
				Malaria	Maceration or infusion	
*Balanites aegyptiaca* (L.) Delile, Zygophyllaceae, G/30/83	Laloub	Tree	Fruit pulp	Diabetes	Infusion	1.03
				Hypertension	Infusion	
				Bilharzia	Infusion	
				Jaundice	Infusion	
*Bauhinia reticulata* DC., Leguminosae, M/50/85	Khroob	Tree	Fruit	Hypertension	Maceration	0.57
			Bark	Jaundice	Infusion	
				Wounds	Powder sprinkle in wound	
*Bergia suffruticosa* (Delile) Fenzl, Elatinaceae, W/10/06	Shagarat almoya	Herb	Aerial part	Eczema	Powder rubbed locally	0.09
*Blepharis linariifolia* Pers., *Acanthaceae*, MA/38/77	Bagail	Herb	Aerial part	Kidney disorders	Maceration	0.87
				Diabetes	Maceration or infusion	
				Wounds	Powder sprinkled in wound	
				Hypertension	Maceration or infusion	
				Toothache	Filling tooth cavity with powder	
				Tonic	Maceration	
*Boswellia papyrifera* (Caill. ex Delile) Hochst., Burseraceae, K/12/96	Tarag tarag	Tree	Bark	Diabetes	Maceration	0.17
				Diarrhoea	Infusion	
				Anaemia	Infusion	
*Calotropis procera* (Aiton) Dryand., Apocynaceae, W/20/94	Ushar	Shrub	Stem	Scorpion sting	Latex squeezed and rubbed locally	0.07
*Carissa spinarum* L., Apocynaceae, W/52/05	Alaly	Shrub	Root	Evil eye	Smoke fumigant	0.93
*Cassia arereh* Delile, Leguminosae, G/36/83	Um Kasho/gafa	Tree	Root	Stomachache	Maceration	0.37
				Malaria	Maceration	
				Toothache	Filling tooth cavity with powder	
				Haematuria	Infusion	
				Evil eye	Smoke fumigant	
*Catunaregam nilotica* (Stapf) Tirven, Rubiaceae, W/111/95	Shagarat Almarfain	Shrub	Bark	Malaria	Maceration	0.37
				Jaundice	Maceration	
				Prostate	Maceration	
*Catunaregam taylorii* (*S.Moore*) *Bridson*, Rubiaceae, K/3/96	Karno	Shrub	Seeds	Kidney disorders	Infusion	0.30
*Chamaecrista nigricans* (Vahl) Greene, Leguminosae, W/101/94	Jub argaly	Herb	Aerial part	Haematuria	Decoction	0.10
*Cissus quadrangularis* L., Vitaceae, G/47/83	Sala sala	Climber	Aerial part	Syphilis	Ash	0.67
				Dandruff	Juice as a head wash	
				Back pain	Powder mixed with sesame oil and rubbed locally	
				Wounds	Poultice applied to wound	
*Cleome gynandra* L., Cleomaceae, W/17/95	Tamalaika	Herb	Leaf	Improve eyesight	Boiled in sour milk	0.23
				Spleen problems	Maceration or infusion	
				Worm expulsion	Decoction	
				Headache	Decoction	
				Rheumatic pain	Decoction	
*Cleome viscosa* L., Cleomaceae, W/10/95	Koda	Herb	Root	Evil eye	Band around arm	0.07
*Clitoria ternatea* L., Leguminosae, M/18/95	Shagarat alyaragan	Herb	Aerial part	Jaundice	Infusion	0.20
				Laxative	Infusion	
				Giardiasis	Infusion	
*Combretum aculeatum* Vent., Combretaceae, K/9/96	Shihait	Shrub	Young branches	Swellings	Poultice applied to swellings	0.07
*Combretum hartmannianum* Schweinf., Combretaceae, G/114/83	Habeel	Shrub	Bark	Rheumatic pain	Smoke fumigant	0.73
*Commiphora gileadensis* (L.) C.Chr., Burseraceae, W/76/95	Gafal	Shrub	Bark	Measles	Decoction	0.07
*Cordia africana* Lam., Boraginaceae, Y/4/010	Gumbail/andrab	Tree	Root	Jaundice	Maceration	0.10
*Coriandrum sativum* L, Apiaceae, Cultivated	Kasbra	Herb	Seed	Foot pain	Poultice	0.02
*Ctenolepis cerasiformis* (Stocks) C.B. Clarke, Cucurbitaceae, O/37/79	Kazaky	Herb	Root	Tonic	Maceration	0.07
*Cymbopogon schoenanthus* (L.) Spreng., Poaceae, G/77/83	Mahraib	Herb	Aerial part	Diabetes	Maceration or infusion	0.50
				Stomachache	Maceration or infusion	
*Cyperus rotundus* L., Cyperaceae, AB/16/94	Siada	Herb	Corm	Kidney stones	Infusion	1.10
				Haematuria	Infusion	
				Worm expulsion	Infusion	
				Headache	Infusion	
				Sexual debility	Maceration or infusion	
*Detarium microcarpum* Guill. & Perr., Leguminosae, G/127/83	Irg abolaila	Tree	Root	Stomachache	Maceration	0.07
*Dicoma tomentosa* Cass., Asteraceae, M/28/95	Um senainat	Herb	Root	Jaundice	Maceration or infusion	0.07
*Dichrostachys cinerea* (L.) Wight & Arn., Leguminosae, K/5/96	Kadad	Tree	Root and fruit	Jaundice	Maceration or infusion	0.30
*Drimia maritima* (L.) Stearn, Asparagaceae, Y/17/015	Baroug/galb albarida	Herb	Bulb	Sexual debility	Maceration	0.17
				Snake bite	Juice rubbed on place of bite	
*Echinops longifolius* A. Rich., Compositae, G/100/83	Irg agrab	Herb	Root	Scorpion sting	Fresh crushed roots rubbed locally	0.07
*Eucalyptus camaldulensis* Dehnh., *Myrtaceae*, Cultivated	Kafoor	Tree	Leaf	Toothache	Filling tooth cavity with powder	0.07
*Fagonia cretica* L., Zygophyllaceae, W/121/95	Um shuwaika	Herb	Aerial part	Skin allergy	Poultice	0.03
*Geigeria alata* Benth. & Hook.f. ex Oliv. & Hiern, Compositae, O/2/81	Gadad	Herb	Aerial part	Diabetes	Infusion	1.67
				Kidney disorders	Infusion	
				Hypertension	Infusion	
				Stomachache	Infusion	
*Grewia flavescens* Juss., Malvaceae, G/42/83	Hilo/khakasan	Shrub	Fruit	Anaemia	Maceration and mixed with nisha	0.63
*Grewia tenax* (Forssk.) Fiori, Malvaceae, G/105/83	Gudaim	Shrub	Bark	Wounds	Poultice applied to wounds	0.63
			Fruit	Anaemia	Maceration and mixed with nisha	
*Grewia villosa* Willd., Malvaceae, G/11/83	Gargadan	Shrub	Bark	Wounds	Poultice applied to wounds	0.67
				Anaemia	Maceration and mixed with nisha	
				Eye infection	Infusion used as an eyewash	
*Guiera senegalensis* J.F.Gmel., Combretaceae, K/35/96	Gubaish	Shrub	Leaf/root	Acid reflux	Mixed with *Ammi visnaga* and sugar and taken as powder	1.87
			Leaf	Malaria	Infusion	
				Kidney disorders	Infusion	
				Diabetes	Infusion	
				Tonic	Infusion	
*Hibiscus sabdariffa* L., Malvaceae, Cultivated	Karkady	Herb	Calyx	Hypertension	Infusion	0.93
				Cold and flu	Infusion	
				Haemorrhoids	Powder applied to the anus	
*Hydnora abyssinica* A.Br., Hydnoraceae, G/65/83	Dumbo dumbo	Parasite	Root	Stomachache	Powder mixed in yoghourt or ajeen	1.83
				Diarrhoea	Decoction	
				Dysentery	Powder mixed in yoghourt	
*Hyphaene thebaica* (L.) Mart., Arecaceae, K/81/96	Dom	Tree	Fruit	Diabetes	Infusion	0.50
				Diarrhoea	Infusion	
				Kidney disorders	Infusion	
*Jatropha curcas* L., Euphorbiaceae, Y/20/014	Habat almolouk	Shrub	Seed	Sexual debility	Eat with date	0.07
*Khaya senegalensis* (Desr.) A. Juss., Meliaceae, G/173/83	Mahougany	Tree	Bark	Malaria	Maceration	0.93
				Jaundice	Infusion	
*Kigelia africana* (Lam.) Benth., Bignoniaceae, G/8/84	Um shotoor	Tree	Fruit	Breast swellings	Poultice applied at night	1.07
				Rheumatic pain	Roasted and powder mixed with sesame oil and rubbed	
				Leprosy	Powder rubbed locally	
*Lannea fruticosa* (Hochst. ex A. Rich.) Engl., Anacardiaceae, Y/3/010	Layoun	Shrub	Bark	Swellings	Poultice applied to swellings	0.03
*Leonotis nepetifolia* (L.) R.Br., Lamiaceae, M/9/76	Faky bla dawaya	Herb	Aerial part	Evil eye	Smoke fumigant	0.43
*Leptadenia arborea* (Forssk.) Schwein., Apocynaceae, W/8/95	Hadana/shaloub shailingo	Climber	Root	Acid reflux	Maceration	0.30
				Diarrhoea	Maceration	
				Swellings	Poultice applied to swellings	
				Jaundice	Maceration	
*Lepidium sativum* L., Brassicaceae, Cultivated	Habat rashad	Herb	Seed	Kidney stones	Powder taken and water drunk after	0.2
*Leptadenia pyrotechnica* (Forssk.) Decne., Apocynaceae, W/53/06	Mirikh	Shrub	Stem	Rheumatic pain	Smoke fumigant	0.07
*Maerua pseudopetalosa* (Gilg & Gilg-Ben.) DeWolf, Capparaceae, G/107/83	Kurdala	Herb	Root	Diabetes	Masticated then drink water	0.80
				Sexual debility	Masticated then drink water	
				Hypertension	Smoke fumigant	
				Kidney disorders	Infusion	
*Maerua oblongifolia* (Forssk.) A.Rich., Capparaceae, G/21/82	Wad elbarieh/irig mahaba	Shrub	Root	Evil eye/luck	Smoke fumigant	0.23
*Martynia annua* L., Martyniaceae, Y/5/014	Irg agrab/maklab shytan	Herb	Root	Scorpion sting	Fresh crushed roots rubbed locally	0.63
*Mentha spicata* L., Lamiaceae, Cultivated	Nanaa	Herb	Aerial part	Flatulence	Decoction	0.60
*Moringa oleifera* Lam., Moringaceae, Y/17/014	Moringa	Shrub	Seed	Back pain	Powder mixed with sesame oil and salt and rubbed	0.20
			Leaf	Fatigue	Infusion	
*Nigella sativa* L., Ranunculaceae, Cultivated	Kamoon aswad	Herb	Seed	Articulation pain	Powder mixed with sesame oil	1.20
				Stomachache	Maceration	
				Headache	Infusion	
			Aerial part	Jaundice	Infusion	
*Oldenlandia uniflora* L., Rubiaceae, Y/12/015	Shagarat albahag	Herb	Root	Leprosy	Powder rubbed locally	0.13
*Opuntia ficus-indica* (L.) Mill., Cactaceae, W/34/95	Teen ahawky	Shrub	Latex	Dandruff	Boil in sesame oil and rub hair skin before wash	0.07
*Pennisetum glaucum* (L.) R.Br., Poaceae, Cultivated	Duchen	Herb	Seed	Measles	Powder mixed with milk of black goat and drunk and as body wash	0.10
				Sexual debility	Powder prepared as pudding and eaten	
*Plicosepalus acaciae* (Zucc.) Wiens & Polhill, Loranthaceae, W/161/95	Enaba	Parasite	Bark	Evil eye	Powder mixed with gum and wax of black goat	0.07
			Seed	Repels insect from ear	Smoke fumigant	
*Rhynchosia minima* (L.) DC., Leguminosae, W/44/95	Adan alfar/shgr dabib	Herb	Root	Snake bite	Fresh crushed roots rubbed on place of bite	0.50
*Sarcocephalus latifolius* (Sm.) E.A.Bruce, Rubiaceae, K/14/96	Um dimy	Shrub	Root or fruit	Malaria	Maceration	2.067
				Jaundice	Maceration	
				Diabetes	Infusion	
			Fruit pulp	Stomachache	Maceration	
				Acid reflux	Infusion	
*Sclerocarya birrea subsp. caffra* (Sond.) Kokwaro, Anacardiaceae, G/92/83	Hommaid	Tree	Bark	Jaundice	Decoction	0.93
				Diarrhoea	Maceration or infusion	
				Stomachache	Maceration or infusion	
*Senna italica* Mill., Leguminosae, W/14/95	Sena sena	Herb	Fruit	Dysentery	Maceration or infusion	0.60
				Laxative	Maceration or infusion	
				Acne	Powder rubbed locally	
*Senna obtusifolia* (L.) H.S.Irwin & Barneby, Leguminosae, G/39/83	Kawal	Herb	Leaf and seed	Jaundice	Decoction	0.63
*Senna occidentalis* (L.) Link, Leguminosae, W/63/95	Bun balash/soreib	Shrub	Seed	Diabetes	Infusion	0.13
				Eczema	Powder rubbed locally	
*Setaria acromelaena* (Hochst.) T.Durand & Schinz, Poaceae, Y/7/015	Um lisaig	Herb	Root	Evil eye	Smoke fumigant	0.07
*Solanum dubium* Dunal, Solanaceae, W/16/95	Um gibin	Shrub	Root	Jaundice	Decoction	0.07
*Sonchus cornutus* Hochst. ex Oliv. & Hiern, Compositae, W/12/03	Moleata	Herb	Leaf	Malaria	Infusion	0.200
				Diabetes	Infusion	
*Stylochiton grandis* N.E.Br., Araceae, O/6/79	Marouro	Herb	Root	Scorpion sting	Fresh crushed roots rubbed on place of bite	0.93
*Striga hermonthica* (Delile) Benth., Orobanchaceae, Y/42/014	Boda	Parasite	Aerial part	Menstrual cramps	Maceration	0.93
				Diabetes	Maceration	
*Strychnos spinosa* Lam., Loganiaceae, W/66/95	Umm bekhesa	Tree	Fruit	Hypertension	Eaten	0.07
*Tamarindus indica* L., Leguminosae, G/132/83	Aradaib	Tree	Fruit pulp	Malaria	Macerated with lemon, calyx of *Hibiscus sabdariffa* and pods of *Acacia nilotica subsp. adstringens*	0.30
			Bark	Evil eye	Maceration	
			Seed	Kidney disorders	Infusion	
*Terminalia brownii* Fresen., Combretaceae, M/8/79	Sobag	Tree	Bark	Jaundice	Decoction until water gets yellow in colour	0.30
				Rheumatic pain	Smoke fumigant	
				Wounds	Poultice applied to wound	
*Terminalia laxiflora* Engl., Combretaceae, G/102/83	Daroat	Tree	Bark	Malaria	Maceration	0.07
*Tephrosia uniflora* Pers., Leguminosae, S/17/97	Tor farid	Herb	Leaf	Urine retention	Maceration	0.50
				Prostate	Maceration	
*Thymus vulgaris* L., Lamiaceae, Purchsed	Zaatr	Herb		Rheumatic pain	Mixed with olive oil and rubbed	0.07
*Tinospora bakis* (A. Rich.) Miers, Menispermaceae, Y/7/014	Bun balash/irg alhagar	Climber	Root	Swellings	Poultice applied to swelling	0.83
				Snake bite	Maceration	
				Stomachache	Maceration	
				Malaria	Macerated with clove	
				Diabetes	Maceration or infusion	
				Evil eye	Smoke fumigant	
*Tribulus terrestris* L., Zygophyllaceae, W/83/95	Diraisa	Herb	Root	Kidney disorders	Maceration	0.70
				Diabetes	Maceration	
*Trigonella foenum-graecum* L*.*, Leguminosae, Cultivated	Hilba	Herb	Seed	Uterus inflammation	Mixed with curcuma, black cumin and bee honey	0.63
				Swellings	Poultice applied to swellings	
				Foot pain	Cataplasm	
*Vangueria madagascariensis* J.F.Gmel., Rubiaceae, W/45/95	Kir kir	Tree	Fruit	Diabetes	Maceration	0.10
				Kidney disorders	Maceration	
				Hypertension	Maceration	
*Ximenia americana* L., Olacaceae, Y/17/014	Jabl fungur	Tree	Bark	Rheumatic pain	Mixed with salt, fruit of *Acacia nilotica subsp. adstringens* and sesame oil and rubbed	0.07
*Ziziphus spina-christi* (L.) Desf., Rhamnaceae, W/122/95	Sidir	Tree	Leaf	Evil eye	Maceration and drunk or as body wash	0.73
			Fruit	Stomachache	Sousing the mesocarp	
			Bark	Dysentery	Decoction with atroon	

### Habitat of the plants

**Fig. 2:**
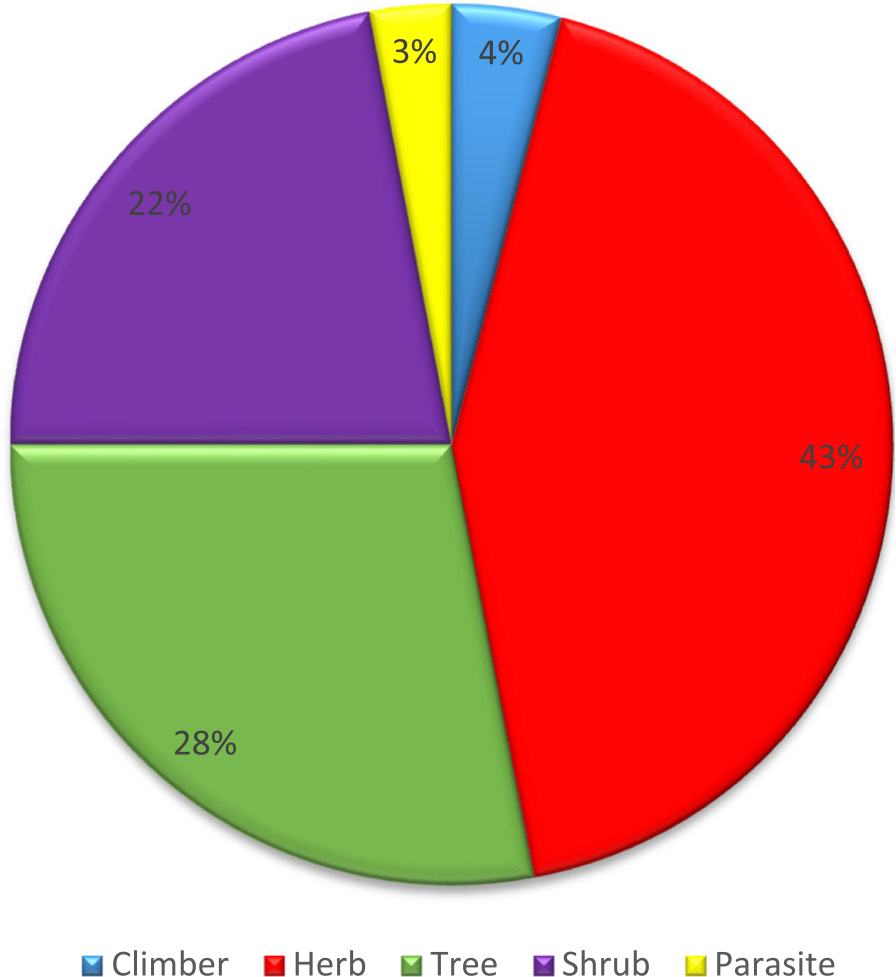
Habitat of medicinal plants in the study area

Analysis of data based on their habitat showed that the reported species belong to herbs (43%), trees (28%), shrubs (22%), climbers (4%) and parasites (3%) (Fig. 2). The majority of medicinal plants are collected from the wild, and only 11% are cultivated or purchased (0.01%) from the market (Table 1).

### Parts of medicinal plants used

**Fig. 3:**
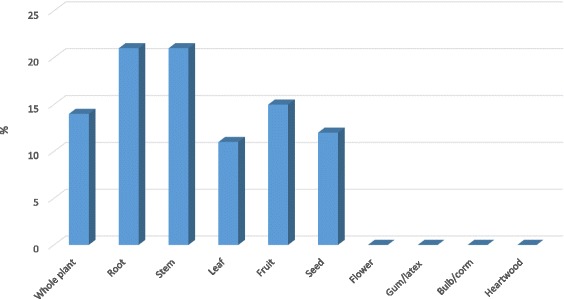
Percentage of plant parts used

Data on different plant parts used in traditional medicine are indicated in Fig. 3. Those that are used the most were the root and stem (21% each) followed by the fruit (15%), whole plant (14%), seed (12%), leaf (11%), gum/latex, bulb/corm and heartwood (0.02%) and flower (0.01%) respectively. There are cases where different parts of the same plant are being used for the treatment of different diseases.

### Method of preparation

A majority of remedies are administered orally (67%) where infusion (36%) and maceration (32%) are the most used methods. Some prescriptions can be prepared by both methods: infusion or maceration represented 13%, while decoction represented 11% of preparations. Dried powder or freshly collected plant parts are also used. Other prescriptions are used externally (33%) and applied as dry powder (29%), rub (23%), smoke (23%), poultices (20%) or as a wash (6%) (Table 2). Most of these preparations use water as a solvent extractor. Some herbalists used other adjuvants like honey, sugar, salt, milk, sour milk, yoghurt, ajeen (fermented dough), nisha (light porridge), atroon (sodium bicarbonate), bee wax, wax of goat and olive and sesame oil.

**Table 2:** Mode of preparations of medicinal plants in the study area

Oral		External	
Decoction	15 (11%)	Smoke	15 (23%)
Infusion	48 (36%)	Poultice	13 (20%)
Maceration	43 (32%)	Rubbed	15 (23%)
Infusion/maceration	17 (13%)	Wash	4 (06%)
Powder	12 (09%)	Powder	19 (29%)
Total	135 (67%)	Total	66 (33%)

### Medicinal plants used in combination

For the treatment of particular ailment, sometimes herbalists used more than one plant. For example, *Allium sativum* bulb is mixed with *Zingiber officinale* rhizome and applied to the anus for the treatment of haemorrhoids. A potion is prepared from the seed of *Trigonella foenum-graecum*, curcuma, *Negilla sativa* and bee honey for the treatment of uterus inflammation. Root of *Tinospora bakis* is mixed with *Syzygium aromaticum* (clove) for the treatment of malaria. Atroon is added to some preparations like those of *Ziziphus spina-christi* and *Acacia oerfota* for the treatment of dysentery and toothache respectively.

### Quantitative analyses of ethnomedicinal data

#### Informant consensus factor

Fifteen ailment categories were identified. The ICF was calculated for each ailment category, and the range was from 0.50 to 0.91 (Table 3). The highest ICF (0.91) was reported for poisonous animal bites with 8 species and 77 use reports, followed by urinary system diseases (0.89) with 17 species and 156 use reports, blood system disorders (0.88) with 14 species and 116 use reports and gynaecological diseases (0.87) with 12 species and 86 use reports. The highest ICF for poisonous animal bites can be probably related to the hard and dangerous environmental conditions. The category of plants used for treatment of eye diseases has the lowest degree of consensus (0.50) where only three informants mentioned ailments in this category.

**Table 3:** Diseases based on categories and informant consensus factor (ICF)

	*N*_t_	*N*_ur_	ICF
Respiratory system diseases	8	31	0.77
Blood system disorders	14	116	0.88
Urinary system	17	156	0.89
Gynaecological diseases	12	86	0.87
Muscoloskeletal system	15	90	0.84
Dermatology	19	64	0.71
Digestive system disorders	48	292	0.84
Parasite infections	22	126	0.83
Endocrinological system (diabetes)	16	89	0.83
Abnormalities	9	45	0.82
Poisonous animal bites	8	77	0.91
Pain	10	43	0.76
Eye diseases	2	3	0.50
General health	4	13	0.75
Envy eye	12	89	0.86

Respiratory system diseases: cold, cough, flu, asthma, measles and ear infection. Blood system disorders: hypertension, anaemia and spleen problems. Urinary system: kidney disorders, kidney stones, urine retention and haematuria. Gynaecological diseases: uterus inflammation, menstruation, syphilis, postpartum, prostate and sexual weakness. Muscoloskeletal system: rheumatism, back pain and foot pain. Dermatology: skin diseases, skin allergy, wounds, eczema, leprosy and dandruff. Digestive system disorders: stomachache, flatulence, acid reflux, diarrhoea, haemorrhoids, dysentery, laxative and jaundice. Parasite infections: bilharzia, malaria, giardiasis and helminthiasis. Endocrinological system: diabetes. Abnormalities: swellings. Poisonous animal bites: scorpion sting and snake bite. Pain: headache and toothache. Eye diseases: improved eyesight, eye infection. General health: tonic

*N*_*t*_ number of taxa, *N*_*ur*_ number of use reports

#### Most frequently cited plant species and medicinal uses

In this study, the most cited plants, those that had at least 20 or more citations for specific ailment, were *Guiera senegalensis* (57 citations) mainly used for the treatment of malaria (22 citations) and kidney disorders (20 citations). This is followed by *Hydnora abyssinica* (55 citations) used in the treatment of gastrointestinal system diseases (mainly for diarrhoea and dysentery (40 citations), *Geigeria alata* (50 citations) used mainly for the treatment of diabetes (20 citations) and hypertension (17 citations), *Kigelia africana* (32 citations) with 28 citations for the treatment of breast swellings and *Carissa spinarum* (28 citations) for envy eye.

#### Medicinal plants and the associated knowledge

**Fig. 4:**
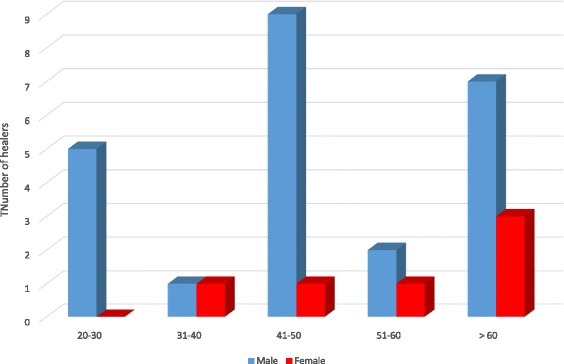
Age group distribution of the traditional healers interviewed

Thirty healers (24 male and 6 female) were interviewed and divided into five different age groups (20–30, 31–40, 41–50, 51–60 and > 60). Analysis of the result on ages of healers revealed that the most dominant age of men is 41–50 while for women which were few in number is > 60 (Figs. 3 and 4).

## Discussion

In this study, the most cited plants, *Guiera senegalensis*, *Hydnora abyssinica*, *Geigeria alata*, *Kigelia africana* and *Carissa spinarum*, were previously reported with the same traditional uses in ethnobotanical studies from other regions of Sudan. For example, *Guiera senegalensis* was reported by EL-Kamali [3] and Suleiman [21] for the treatment of malaria. *Hydnora abyssinica* (*H. johannis*) for the treatment of diarrhoea and dysentery and *Kigelia africana* for the treatment of breast swellings were also reported by Musa et al. [22]. *Geigeria alata* for the treatment of diabetes was reported by EL-Kamali [3] and Suleiman [21]. *Carissa spinarum* (*C. edulis*) was reported by EL-Kamali [3] for charm and the treatment of madness. *Kigelia africana* was reported by Doka and Yagi [23] for swollen mastitis.

The high frequency of citations of medicinal plants can be explained by the fact that these plants are the best known and have long been used by the majority of informants, representing a source of reliability. In fact, many biological activity and phytochemical evaluation were carried out for these plants. For example, Traore-Keita et al. [24] reported that the chloroform extract of roots of *Guiera senegalensis* exhibited a pronounced antimalarial activity. They isolated two alkaloids, namely, harman and tetrahydroharman, that displayed high antimalarial activity (IC_50_ (50% inhibition) lower than 4 μg/mL) and low toxicity against human leukemia monocytic cell line (THP1). Yagi et al. [25] found that *Hydnora johannis* roots have no activity against bacteria spp. that are mainly responsible of diarrhoea but are rich in phenols. They suggested that the curing potency of the roots of *H. johannis* was not mainly associated with the presence of antibacterial activity agent(s) against bacterial species responsible of dysentery or diarrhoea but might be attributed to the role of tannins in reducing the effect through denaturing the proteins by the formation of protein tannate, thereby causing the intestinal mucosa to become more resistant, reducing the intestinal transit and by acting as a barrier against toxin exerted by bacteria. The antidiabetic potential of *Geigeria alata* root was evaluated, and diabetic rats dosed with 250 mg/kg of aqueous methanolic extract were found to have significantly (*p* < 0.05) decreased blood glucose level closer to that of non-diabetic rats and improved β-cell function and antioxidant status [26]. *Kigelia africana* was found to suppress the breast MCF7 [27], human colon adenocarcinoma (Caco-2), human embryonic kidney (HEK-293) [28] and HeLa cervical cancer cell proliferation [29].

### Comparative review of traditional usages of reported species with previous studies from Sudan

A comparative review with previous reports [3, 21–23, 30–33] from different parts of Sudan was performed to identify the new medicinal plants and new uses reported in this study (Table 4). The plants reported by Suleiman [21] for traditional plants used by communities of Northern Kordofan region included a total of 44 plant species with 22 species with same traditional uses which were reported also in this study, while 2 species, *Blepharis linariifolia* and *Catunaregam nilotica* (*Xeromphis nilotica*, *Randia nilotica*), were reported with different uses. EL-Kamali [3] reported 48 plant species for traditional plant uses in North Kordofan too with 15 species with same traditional uses which were reported also in this study and 5 species, *Acacia nilotica subsp. adstringens*, *Aristolochia bracteolate*, *Cissus quadrangularis*, *Dichrostachys cinerea* and *Sarcocephalus latifolius* (*Nauclea latifolia*), with different uses. Doka and Yagi [23] reported 49 plant species for traditional plant uses in West Kordofan with 16 species with same traditional uses which were reported also in this study, and 9 species were reported in this study with different uses; these included *Acacia senegal*, *Acacia seyal*, *Arachis hypogaea*, *Balanites aegyptiaca*, *Cissus quadrangularis*, *Combretum aculeatum*, *Grewia flavescens*, *Tamarindus indica* and *Catunaregam nilotica*. Musa et al. [22] reported 53 plant species for traditional plant uses in the Blue Nile State, southeastern Sudan, with 18 species with same traditional uses which were reported in this study and 13 species with different uses: *Acacia senegal*, *Acacia seyal*, *Anogeissus leiocarpus*, *Carissa spinarum* (*C. edulis*), *Cissus quadrangularis*, *Grewia villosa*, *Lannea fruticose*, *Piliostigma reticulatum*, *Senna occidentalis*, *Strychnos spinose*, *Tephrosia uniflora*, *Terminalia laxiflora* and *Ximenia americana*. Moreover, El Ghazali et al. [30–33] in their books of Sudanese medicinal plants documented some of these plants for the same or very similar usages. In fact, there are 99 new traditional uses for some previously reported medicinal plants. For example, the whole plant of *Striga hermonthica* was previously reported to treat diabetes, but in this study, it is used also for menstrual cramps. The fruit of *Senna occidentalis* is reported to treat eczema beside its common use as a laxative. *Plicosepalus acaciae* is commonly used to enhance wound healing and as a lactagogue, but in this study, the smoke fumigant of the seeds is reported to repel insect from ear.

**Table 4:** Comparative review of traditional usages of reported species with previous studies from Sudan

Plant name	Disease treated	Suleiman [18]	EL-Kamali [2]	Koda and Yagi [20]	Musa et al. [19]	El Ghazali et al. [27–30]
*Abrus precatorius*	Spleen problems					Snake bite^1^
						Headache^4^
*Acacia nilotica subsp. adstringens*	Stomachache	Cold and flu and pharyngitis	Hypertension	Cough	Phlegmatic cough	Cold and flu^3, 4^
	Cold and flu	Tonsillitis			Furuncles	Tonsillitis^2^
	Wounds	Fever			Malaria	
		Measles				
		Hypertension				
		Catarrh				
		Antiseptic				
*Acacia oerfota*	Back pain	Antirheumatic		Tooth cavity	Toothache	Swellings^4^
	Swellings				Headache	Scorpion sting^4^
	Snake bite				Snake bite	
	Toothache					
*Acacia senegal*	Haematuria	Rheumatoid arthritis		Giardiasis	Kidney problems	
	Toothache	Heartburn				
*Acacia seyal*	Rheumatic pain			Leprosy	Diarrhoea	Diarrhoea^2^
				Bleeding	Dysentery	Dysentery^2^
*Acanthorrhinum ramosissimum*	Evil eye					
*Adansonia digitata*	Giardiasis	Dysentery	Fever	Pain after birth	Malaria	Stomachache^4^
	Stomachache	Diarrhoea	Diarrhoea		Diarrhoea	
		Stomachache			Dysentery	
		Fever				
		Kidney stones		Diarrhoea		
*Albizia anthelmintica*	Anthelmintic	Anthelmintic	Anthelmintic	Anthelmintic		Stomachache^4^
	Wounds					
	Stomachache					
	Jaundice					
*Allium sativum*	Haemorrhoids					Haemorrhoids^5^
*Anastatica hierochuntica*	Postpartum					
*Anogeissus leiocarpus*	Toothache	Diabetes			Cough	Cough^1^
	Jaundice	Dysentery			Giardiasis	
	Malaria	Wound			Dysentery	
		Urine retention				
		Malaria				
*Anticharis senegalensis*	Swellings					Swellings^2^
*Arachis hypogaea*	Bilharzia			Scorpion bite		
*Aristolochia bracteolata*	Malaria	Malaria	Scorpion sting		Malaria	Malaria^1^
	Ear infection	HIV-1				Antitumour^3^
	Headache	Scorpion sting				Scorpion sting^4^
		Ear infection				
		Wounds				
		Toothache				
		Headaches				
*Azadiracta indica*	Rheumatic pain		Antipyretic		Malaria, fever,	Fever^2^
	Malaria		Backache		Jaundice	Scorpion sting^3^
						Snake bite^3^
						Intestinal spasm^3^
						Anthelmintic^4^
						Constipation^4^
*Balanites aegyptiaca*	Diabetes	Stomachache	Antispasmodic	Malaria		Diabetes^2^
	Hypertension	Anthelmintic	Stomach pain	Kidney disorders		Constipation^2^
	Bilharzia	Dysentery	Diabetes			Constipation^3^
	Jaundice	Constipation				Bilharzia^3^
		Jaundice				Wound^3^
		Diabetes				
						Syphilis^2^
*Bergia suffruticosa*	Eczema					Leucoderms^2^
*Blepharis linariifolia*	Kidney disorders	Swellings	Stomach pain	Urine retention		Stomach pain^4^
	Diabetes		Kidney stone			Bilharzia^4^
	Wounds					
	Hypertension					
	Toothache					
	Tonic					
*Boswellia papyrifera*	Diabetes			Dysentery	Bilharzia	Jaundice^4^
	Diarrhoea			Respiratory infections	Diarrhoea, dysentery	
	Anaemia					
*Calotropis procera*	Scorpion sting	Scorpion sting	Haemorrhoids	Scorpion sting		Wounds^2^
	Wounds	Haemorrhoids	Scorpion sting	Rheumatic pain		Rheumatic pain^2^
		Rheumatic pain				Scorpion sting^4^
		Wounds				Jaundice^4^
*Carissa spinarum* (Syn. *C. edulis*)	Evil eye		Kidney disorders		Treating rashes	Skin lesions^1^
			Charm and madness			Stomachache^4^
						Headache^4^
						Cough^4^
						Anthelmintic^4^
*Cassia arereh*	Stomachache				Stomachache	
	Malaria				Diarrhoea	
	Toothache				Evil eye	
	Haematuria					
	Evil eye					
*Catunaregam nilotica* (Syn. *Randia nilotica*, *Xeromphis nilotica*)	Malaria	Swellings	Swellings	Rabies	Measles	Jaundice^4^
	Jaundice	Tonsillitis	Tonsillitis		Toothache	Anthelmintic^4^
	Prostate	Dandruff	Jaundice			Rabies^4^
			Dandruff			
*Catunaregam taylorii*	Kidney disorders					
*Chamaecrista nigricans* (Syn. *Senna nigricans*)	Haematuria					Stomachache^4^
*Cissus quadrangularis*	Syphilis	Syphilis	Haemorrhoids	Syphilis	Acne	Pruritus^1^
	Dandruff	Asthma		Leprosy	Evil eye	Scorpion sting^4^
	Back pain	Haemorrhoids		Snake bite		Stomachache^4^
	Wounds	Snake bite				Joint pain^4^
		Tuberculosis				
*Cleome gynandra* (Syn. *Gynandropsis gynandra*)	Improve eyesight					
	Spleen problems					
	Worm expulsion					
	Headache					
	Rheumatic pain					
*Cleome viscosa L.*	Evil eye					
*Clitoria ternatea*	Jaundice				Constipation	Constipation^1^
	Laxative					
	Giardiasis					
*Combretum aculeatum*	Swellings			Snake bite		Wound^3^
						Constipation^4^
						Tuberculosis^4^
*Combretum hartmonnianum*	Rheumatic pain					Jaundice^3, 4^
*Commiphora gileadensis*	Measles	Antirheumatic				
		Typhoid fever				
*Cordia africana*	Jaundice	Cuts, burns and wounds	Cuts, wounds and burns			
*Coriandrum sativum*	Foot pain					Hypertension^5^
*Ctenolepis cerasiformis*	Tonic					
*Cymbopogon schoenanthus*	Diabetes	Antispasmodic				Stomachache^2^
	Stomachache	Stomachache				
		Gout				
		Helminthiasis				
		Inflammation of prostate				
*Cyperus rotundus*	Kidney stones					
	Haematuria					
	Worm expulsion					
	Headache					
	Sexual debility					
*Detarium microcarpum*	Stomachache		Rheumatism			
*Dichrostachys cinerea*	Jaundice		Wounds		Stomachache	
	Asthma				Diarrhoea	
	Evil eye				Toothache	
					Jaundice	
					Sexual debility	
*Dicoma tomentosa*	Jaundice					Toothache^1^
						Febrifuge^1, 4^
						Mumps^3^
*Drimia maritima*	Sexual debility					
	Snake bite					
*Echinops longifolius*	Scorpion sting					
*Eucalyptus camaldulensis*	Toothache					
*Fagonia cretica*	Skin allergy			Skin allergy		Stomachache^2^
						Muscular pain^3^
*Geigeria alata*	Diabetes	Antispasmodic	Diabetes			Stomachache^2^
	Stomachache	Stomachache	Antispasmodic			Epilepsy^3^
	Kidney disorders	Intestinal complaints	Intestinal complaints			
	Hypertension	Anthelmintic	Hypertension			
		Diabetes	Cough			
		Hypertension				
		Cough				
*Grewia flavescens*	Anaemia			Stomach disorders		Tuberculosis^4^
				Leprosy		
*Grewia tenax*	Wounds	Tonsillitis, throat infections				Tonsillitis^2^
	Anaemia	Anaemia				Swellings^2^
		Malaria				Jaundice^3^
		Tonic				Trichoma^3^
*Grewia villosa*	Wounds	Wounds			Cancer	Constipation^1^
	Eye infection	Syphilis				
		Arthralgia				
		Eye ache				
*Guiera senegalensis*	Acid reflux	Jaundice	Stomach pain			Leprosy^1, 4^
	Malaria	Antipyretic	Jaundice			Antipyretic^2, 3, 4^
	Kidney disorders	Antispasmodic	Malarial fever			Leprosy^3^
	Diabetes	Diarrhoea	Antispasmodic			Vomiting^4^
	Tonic	Leprosy	As a tonic			
		Diabetes				
		Hypertension				
		Malarial fever				
		Wound				
*Hibiscus sabdariffa*	Hypertension	Cough	Snake bite			
	Cold and flu	Headache	Scorpion sting			
	Hypertension	Haematuria	Haemorrhoids			
	Haemorrhoids	Hypertension	Headache			
		Fever				
		Snake bite				
		Scorpion sting				
*Hydnora abyssinica* (Syn. *H. johannis*)	Stomachache				Cholera	Dysentery^2^
	Diarrhoea				Diarrhoea	Tonsillitis^2^
					Dysentery	Swellings^2^
	Dysentery				Evil eye	
*Hyphaene thebaica*	Diabetes					Spleen problems^5^
	Diarrhoea					Stomachache^5^
	Kidney disorders					Wound^5^
*Jatropha curcas*	Sexual debility		Laxative		Giardia	
					Jaundice	
					Malaria	
					Fever	
*Khaya senegalensis*	Malaria	Malarial fever	Malarial fever		Malaria	Headache^4^
	Jaundice	Syphilis	Asthma		Diabetes	Stomachache^4^
		Taeniacide	Intestinal complaints			Dysentery^4^
		Hepatic inflammation				
		Jaundice				
		Trachoma				
		Enterogastritis				
*Kigelia africana*	Breast swellings			Swollen mastitis	Breast tumour	
	Rheumatic pain				Hypertension	
	Leprosy				Diabetes	
*Lannea fruticosa*	Swellings				Dysentery	
					Wound	
*Leonotis nepetifolia*	Evil eye					Swellings^4^
						Stomachache^4^
*Leptadenia arborea*	Acid reflux	Jaundice		Jaundice		Snake bite^3^
	Diarrhoea	Dandruff		Dandruff		Gonorrhoea^4^
	Swellings					Swellings^4^
	Jaundice					
*Leptadenia pyrotechnica*	Rheumatic pain	Antirheumatic	Rheumatism			
		Sciatica				
		Urine retention				
*Lepidium sativum*	Kidney stones					Swellings^5^
*Maerua pseudopetalosa*	Diabetes					
	Sexual debility					
	Hypertension					
	Kidney disorders					
*Maerua oblongifolia*	Evil eye/luck					Snake bite^2^
*Martynia annua*	Scorpion sting					
*Mentha spicata*	Flatulence					Flatulence^5^
*Moringa oleifera*	Back pain					
	Fatigue					
*Nigella sativa*	Articulation pain					Diabetes^5^
	Stomachache					Hypertension^5^
	Headache					Stomachache^5^
	Jaundice					
*Oldenlandia uniflora*	Eczema					
	Leprosy					
*Opuntia ficus-indica*	Dandruff					
*Pennisetum glaucum*	Measles					Rheumatic pain^5^
	Sexual debility					
*Plicosepalus acaciae*	Evil eye					Lactagogue^2^
	Repels insect from ear					Wound^2^
*Piliostigma reticulatum*	Hypertension				Snake bite	Snake bite^1^
	Jaundice					
	Wounds					
*Rhynchosia minima*	Snake bite					Anti acid^1^
*Sarcocephalus latifolius* (Syn. *Nauclea latifolia*)	Malaria	Malarial fever	Headache, cough			Tapeworms^1^
	Jaundice	Headache	Antihypertensive			Dysentery^4^
	Diabetes	Cough	Kidney disorders			Cough^4^
	Stomachache	Hypertensive				Abdominal pain^4^
	Acid reflux	Kidney disorders				
		Dysentery				
		Abdominal pain				
*Sclerocarya birrea subsp. caffra*	Jaundice	Dysentery	Suleiman (2015) [21]		Dysentery	Stomachache^4^
	Diarrhoea	Diarrhoea			Diarrhoea	Diarrohea^4^
	Stomachache	Diabetes				
*Senna italica*	Dysentery			Constipation		Rheumatic pain^3^
	Laxative					
	Eczema					
*Senna occidentalis*	Diabetes	Backache	Backache	Diabetes	Jaundice	Jaundice^3^
	Eczema	Hypertension	Hypertension	Gonorrhoea		
		Malaria		Intestinal ulcer		
		Dysentery				
		Jaundice				
*Senna obtusifolia*	Jaundice	Jaundice	Jaundice		Jaundice	Constipation^4^
	Eczema					Ringworm^4^
						Wound^4^
*Setaria acromelaena*	Evil eye					
*Solanum dubium*	Jaundice					
*Sonchus cornutus*	Malaria					
	Diabetes					
*Striga hermonthica*	Menstrual cramps		Diabetes			
	Diabetes					Leukoderma^3^
*Strychnos spinosa*	Hypertension			Hypertension	Sexual debility	
*Stylochiton grandis*	Scorpion sting					Scorpion sting^2^
*Tamarindus indica*	Malaria	Malaria		Food poisoning	Malaria	Malaria^4^
	Kidney disorders	Malaria fever		Toothache	Fever	Constipation^4^
	Evil eye	Cold and flu			Stomachache	
		Jaundice			Wound	
		Constipation				
*Tephrosia uniflora*	Urine retention				Diarrhoea	Headache^1, 4^
	Prostate					Tonic^4^
*Terminalia brownii*	Jaundice					Diabetes^1^
	Rheumatic pain					Cough^2^
	Wound					
*Terminalia laxiflora*	Malaria				Cough, tonic	
*Thymus vulgaris*	Rheumatic pain					Flatulence^5^
*Tinospora bakis*	Swelling		Abdominal pain			Wound^1^
	Snake bite					
	Stomachache					
	Malaria					
	Diabetes					
	Evil eye					
*Tribulus terrestris*	Kidney disorders					
	Diabetes					
*Trigonella foenum-graecum*	Uterus inflammation					Swellings^5^
	Swellings					Haemorrhoids^5^
	Foot pain					
*Vangueria madagascariensis*	Diabetes				Diabetes	
	Kidney disorders					
	Hypertension					
*Ximenia americana*	Rheumatic pain			Rheumatic pain		Measles^1^
*Ziziphus spina-christi*	Stomachache	Swellings	Antispasmodic		Stomachache,	Swellings^2^
	Dysentery	Antispasmodic	Fever		Dysentery	Constipation^2^
	Evil eye	Constipation			Diarrhoea	Intestinal spasm^3^
		Gonorrhoea			Malaria	Stomachache^4^
					Urine retention	Gonorrhoea^4^

New species and new uses for species are reported for the first time in this study. For example, *Anastatica hierochuntica*, *Ctenolepis cerasiformis*, *Echinops longifolius*, *Cleome gynandra*, *Maerua pseudopetalosa*, *Martynia annua*, *Oldenlandia uniflora*, *Opuntia ficus-indica*, *Solanum dubium*, *Sonchus cornutus*, *Tribulus terrestris* and *Drimia maritima* were not being mentioned in any previous study for the traditional Sudanese medicine. *Acanthorrhinum ramosissimum*, *Cleome viscosa* and *Setaria acromelaena* which were used for evil eye were also reported for the first time.

The majorities of the healers declared that they had learned about medicinal plants from their parents or grandparents. The lack of systematic documentation for medicinal plant knowledge which appears to occur in many parts of the world may contribute to the loss of this knowledge, particularly for plants that are neglected or non-preferred [34–36].

## Conclusion

The number of medicinal plants reported in this paper reflects evidence that the Algoz area harbours a high diversity of medicinal plants that will continue to play an important role in the healthcare system in the study area. Evaluation of their claimed pharmacological potential efficacy and toxicity profile is essential. Moreover, the present study could contribute in conserving such rich heritage and providing precious information as a contribution through writing the Sudanese pharmacopeia.

Conservation of this traditional knowledge is very important. The progressing mass destruction of wild vegetation for various purposes may accelerate the disappearance of medicinal plants. This in turn may have profound consequences on the roles of traditional medicine on human health. Furthermore, the drop in the availability of raw materials due to the depletion of natural resources affects the discovery of potential drugs [37]. Thus, raising community awareness about conservation and sustainable utilization of the traditional medicinal plants is a vital part for the entire plant biodiversity [22]. Modern biotechnical approaches like genetic engineering, micropropagation via tissue encapsulation of propagules, tissue culture and fermentation should be applied to improve yield and modify the potency of medicinal plants [38].

## Abbreviations

ICF: Informant consensus factor
UV: Use value
